# Response Surface Methodology for the Optimization of Preparation of Biocomposites Based on Poly(lactic acid) and Durian Peel Cellulose

**DOI:** 10.1155/2015/293609

**Published:** 2015-06-18

**Authors:** Patpen Penjumras, Russly Abdul Rahman, Rosnita A. Talib, Khalina Abdan

**Affiliations:** ^1^Department of Process and Food Engineering, Faculty of Engineering, Universiti Putra Malaysia (UPM), 43400 Serdang, Selangor, Malaysia; ^2^Department of Food Science and Technology, Maejo University, Phrae Campus, Phrae 54140, Thailand; ^3^Department of Food Technology, Faculty of Food Science and Technology, Universiti Putra Malaysia (UPM), 43400 Serdang, Selangor, Malaysia; ^4^Department of Biological and Agricultural Engineering, Faculty of Engineering, Universiti Putra Malaysia (UPM), 43400 Serdang, Selangor, Malaysia

## Abstract

Response surface methodology was used to optimize preparation of biocomposites based on poly(lactic acid) and durian peel cellulose. The effects of cellulose loading, mixing temperature, and mixing time on tensile strength and impact strength were investigated. A central composite design was employed to determine the optimum preparation condition of the biocomposites to obtain the highest tensile strength and impact strength. A second-order polynomial model was developed for predicting the tensile strength and impact strength based on the composite design. It was found that composites were best fit by a quadratic regression model with high coefficient of determination (*R* ^2^) value. The selected optimum condition was 35 wt.% cellulose loading at 165°C and 15 min of mixing, leading to a desirability of 94.6%. Under the optimum condition, the tensile strength and impact strength of the biocomposites were 46.207 MPa and 2.931 kJ/m^2^, respectively.

## 1. Introduction

The agricultural and agrofood industries produce great amounts of wastes, which exhibit a tremendous threat to the environment [1]. These agricultural wastes and by-products cause negative impacts in terms of environmental concern due to the increasing amount of pollutant disposal and in terms of industrial sustainability due to the high costs related to their management [2]. Recovering, recycling, and converting of by-products and wastes into value-added products are the policy to control the stream of organic wastes [3]. Therefore, the development of bio-based material has been studied by many researchers. In recent years, the use of natural plant fibers as a reinforcement material in fiber-reinforced plastics (FRP) has been gaining attention due to its advantages such as renewability, low density, and high specific strength [4, 5]. Several plants, such as kenaf, cotton, wood, bamboo, flax, hemp, sisal, jute, and ramie, are rich in cellulose and have been used to reinforce polymers to produce biocomposite materials [6–9]. In the last decade, however, research focusing on the use of cellulosic waste as filler has grown rapidly. Agrowaste materials such as banana rachis, mulberry bark, soybean, soy hulls [10], wheat straw [10, 11], pineapple leaf fiber [12], barley husk and coconut shell [13], rice husk [14], and sugarcane bagasse [15] have been studied as a resource in the production of cellulose fiber.

Cellulose is an important structural component of all plant materials which confers strength and stability to the plant cell walls [16] and is constantly replenished by photosynthesis [17, 18]. It is organized into microfibrils in the cell wall, interrupted by hemicellulose and surrounded by a lignin matrix. Alkali treatment or mercerization is normally used for preparation of cellulose [19]. The alkali treatment is a process of subjecting natural fibers in an interaction with a strong basic aqueous solution to remove noncellulosic components as waxy materials, lignin, hemicellulose, and impurities [20]. The fiber cell walls swell and become round, thus increasing the strength [19].

Using nano- and microcellulose as reinforcements for epoxy or phenol-formaldehyde resins, polymethylmethacrylate, polypropylene, polystyrene, and styrene copolymers has been extensively studied in the past years [21]. The major advantage of cellulose is that it is a good source of biodegradable material. The biodegradability of blends of thermoplastics, low density polyethylene (LDPE), and high density polyethylene (HDPE) with kenaf cellulose has been studied, showing that cellulose content greater than 30 wt.% led to higher degradation [22]. Generally, commodity plastics are widely used for many applications such as packaging material; however, the plastic materials take very long time for environment decomposition, which represent a serious problem with disposal of plastic waste. Substituting nonbiodegradable polymers with biodegradable ones leads to fully renewable and degradable composites [21]. Among the many biodegradable polymers, poly(lactic acid) (PLA) is considered to be one of the most promising renewable resource-based biopolymer matrices due to its high mechanical properties and easy processability compared to other biopolymers [23]. Moreover, its packaging performance characteristics are closely similar to those of polyethylene terephthalate (PET) [24]. Various studies of PLA reinforced with natural fiber have been reported [4, 21, 25–33]. Although a variety of natural fibers were investigated in detail, the use of durian rind cellulose as reinforcing material in PLA has not yet been explored.

Durian (*Durio zibethinus* Murray) is the most popular fruit in Southeast Asia [34]. The consumption of durian and its products has been phenomenal in the global trade market. The total world harvest of durian is 1.4 Mt, dominated by major producers: Thailand (781 kt), Malaysia (376 kt), and Indonesia (265 kt) [35]. It has been vigorously expanded and entrenched into a multidisciplinary of food processing industries, but only one-third of durian is edible, whereas the seeds and the shell become wastes which generate a large amount of biological waste [34]. In common practice, durian residues are burned or sent to landfills, without regard to the surrounding environment [35]. Suitable methods have to be adopted to utilize the waste for the conversion into upgraded products. Durian rind was found to have a good source of cellulose [36]. In our previous paper [37], we reported that the cellulose obtained from durian peel was 33.12 ± 0.108% which is similar with wheat and barley that consist of cellulose approximately 28–48, 29–51, and 31–45%, respectively, and 26–43% in bamboo cane fiber [38]. Therefore, the use of cellulose from durian rind as the reinforcing material in PLA was investigated in this study.

The effects of input variables (cellulose loading, mixing temperature, and mixing time) on the mechanical properties (tensile strength and impact strength) were studied on surface plots and contour plots. In addition, the optimized conditions of the independent variables to maximize tensile strength and impact strength of composites were also reported in this research.

## 2. Experimental

### 2.1. Materials

Poly(lactic acid) (PLA) (Ingeo biopolymer 2003D food packaging-grade with melting flow index of 6 g/10 min at 210°C; specific gravity of 1.24; >98% purity) was purchased in pellets from Natureworks (USA). Durian peel was collected from Phatthalung Province, Thailand. Reagent-grade sodium hydroxide (NaOH) and acetic acid (CH_3_COOH) and technical-grade sodium chlorite (NaClO_2_) of 80% purity were purchased from Fisher Chemicals Sdn. Bhd. (Malaysia).

### 2.2. Extraction of Cellulose from Durian Peel

**Figure 1:**
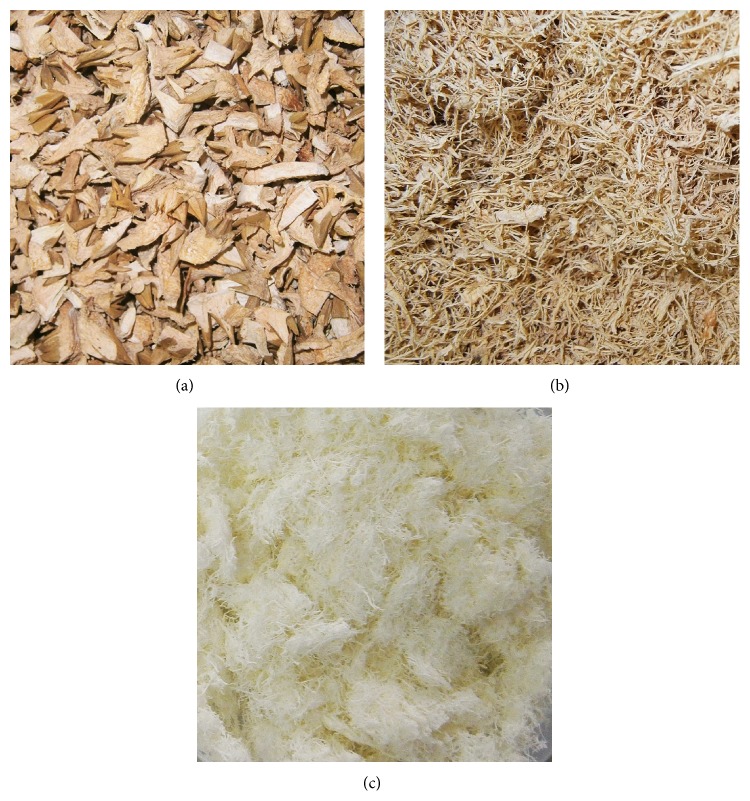
Macroscopic image of (a) durian peel, (b) ground durian peel, and (c) cellulose.

The durian peel was first sun-dried and was then ground as in Figures 1(a) and 1(b). Delignification and mercerization were used to extract cellulose according to Tawakkal et al. [30] with slight modifications. Firstly, delignification was used to produce holocellulose; 20 g of sample was rinsed with tap water to remove dust and was subsequently soaked in a 1,000 mL beaker with 640 mL of distilled water. The beaker was then transferred to a 70°C water bath. Next, 4 mL of CH_3_COOH and 8 g of NaClO_2_ were added to the beaker. Every subsequent hour for total of 5 h, the same amount of CH_3_COOH and NaClO_2_ was added in which lignin was completely separated from the sample. The delignification process was indicated by the color change of samples from brown to white. After that, the sample was left in the water bath overnight. Finally, holocellulose was then filtered, washed, and rinsed with tap water until the yellow color with an odor of chlorine dioxide was removed and the wash water was clear. Secondly, holocellulose was converted to cellulose by mercerization or alkali treatment at room temperature. The holocellulose was added with 80 mL of 17.5% w/v NaOH and the mixture was stirred with a glass rod. Another 40 mL of 17.5% w/v NaOH was added to the mixture every 5 min, three times. The mixture was allowed to sit for 30 min, making the total duration 45 min. Then, 240 mL of distilled water was added to the mixture and allowed to stand for 1 h before filtering. Next, 800 mL of 8.3% w/v NaOH was added to the cellulose for 5 min followed by rinsing with water. The alkaline cellulose was then neutralized by adding 120 mL of 10% v/v acetic acid. The cellulose was subjected to acid treatment for 5 min. Finally, the cellulose was filtered, washed, and rinsed with distilled water until the cellulose residue was free from acid and then dried overnight in a vacuum oven at 80°C. The cellulose obtained is shown as in Figure 1(c). The cellulose was kept in an air tight container at room temperature until analysis.

### 2.3. Characterization of Morphological Feature

The SEM [S-3400N, Hitachi, Japan] images were taken to elucidate the morphologies of the untreated durian peel and cellulose. Samples were mounted on aluminum studs and coated with gold in a vacuum before being observed using a SEM.

### 2.4. Preparation of Biocomposites

The cellulose was ground using grinder and then passed through a sieve (Retsch, AS 200 digit, Germany). The cellulose of sizes 250 to 125 *µ*m was collected. The PLA and cellulose were mixed using an internal mixer (Brabender Plastograph EC, Germany) at a 50 rpm screw speed. Various biocomposites were created at different cellulose loadings, mixing temperatures, and mixing times. To prevent pores' formation, the biocomposites were dried in a convection oven at 80°C for 16 h before further processing. The test specimens were then transferred into a rectangular mould with dimensions of 150 mm × 150 mm. Moulded biocomposites sheets were produced using a hot press machine at 160°C. This process involved 5 min of preheating and 5 min of pressing, followed by 2 min of cooling for tensile testing specimens with 1 mm thickness. Eight minutes of preheating, 7 min of pressing, and 3 min of cooling were used to produce samples for impact testing with 3 mm thicknesses. All biocomposites were packaged in air tight containers and stored at room temperature until analysis.

### 2.5. Mechanical Testing

The tensile strengths were determined using an Instron Universal Testing Machine (Model 5566; USA) at a cross speed of 5 mm/min on specimens with dimensions of 12.7 mm × 63 mm × 1 mm according to ASTM 1882L [39], until tensile failure was detected. Five samples of each biocomposites were tested. The impact test was performed using an Impact Pendulum Tester (Ceast Model 9050) on 2.5 mm notched rectangular specimens with dimensions of 12.7 mm × 63 mm × 3 mm according to ASTM D256 [40]. The Izod method was conducted with a 0.5 J hammer. Seven samples of each biocomposites were tested.

### 2.6. Experimental Design and Statistical Analysis

Response surface methodology (RSM) was used to optimize the conditions for thepreparation of composites. The design of experiment was done using Design Expert 9 (Stat-Ease Inc, USA). Three independent variables were employed by central composite design (CCD). The variables used were cellulose loading (*X*_1_), mixing temperature (*X*_2_), and mixing time (*X*_3_). The design consisted of 20 runs including six axial experiments (levels ±  *α*), eight factorial experiments (levels ± 1), and six replicates in center point. The level of factors and their coding are presented in Table 1. The design matrix is presented in Table 2. The response functions measured were tensile strength and impact strength. A second-order polynomial equation, as a function of *X*, was fitted for each factor as follows:

(1) Y=β0+∑i=13βiXi+∑i=13βiiXi2+∑i=1i<j3βijXiXj,

where *Y* is the estimated response; *β*_0_, *β*_*i*_, *β*_*ii*_, and *β*_*ij*_ are constant coefficients (*β*_0_ a constant, *β*_*i*_ the coefficients for linear terms, *β*_*ii*_ the coefficients for quadratic terms, and *β*_*ij*_ the coefficients for interactive terms); and *X*_1_, *X*_2_, and *X*_3_ are the coded values of the independent variables of cellulose loading (wt.%), mixing temperature (°C), and mixing time (min), respectively. The variance for each factor was partitioned into linear, quadratic, and interactive terms. The lack-of-fit and error components were used in determining the significance of these variables and the suitability of the second-order polynomial function.

### 2.7. Fourier Transform Infrared Spectroscopy (FTIR)

The change in chemical compositions of untreated durian peel, cellulose, and optimal biocomposites was examined by FTIR (Perkin Elmer, Spectrum One FT-IR Spectrometer, USA). All the spectra were recorded in the transmittance mode with a resolution of 4 cm^−1^ in the range of 4000 to 650 cm^−1^. Ten scans were averaged for each sample.

## 3. Results and Discussion

### 3.1. Morphology Analysis

**Figure 2:**
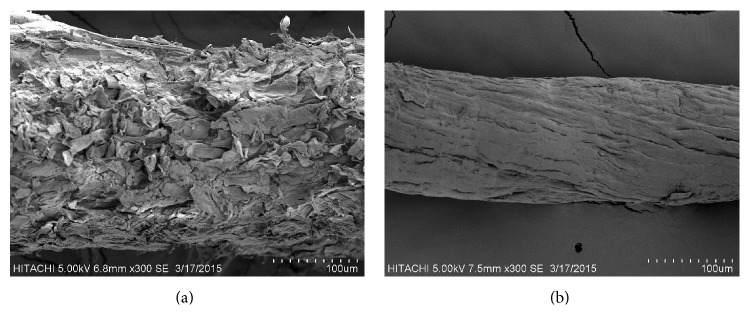
Scanning electron micrograph of (a) untreated durian peel and (b) cellulose.

Figure 2 compares the micrographs of untreated durian peel and cellulose. The micrograph of untreated durian peel shows the amount of noncellulosic components; pectin, lignin, and hemicellulose scattered over the surface [18], which provide the bigger diameter than cellulose. These components were then removed after delignification and alkali treatment. The important consequence of diameter reduction was higher reinforcing ability of the cellulose for composite application because the increasing of aspect ratio (*L*/*d*, *L* is the length and *d* is diameter) [10]. Generally, the minimum aspect ratio for good strength transmission for any reinforcement material is considered at 10 [41]. In our previous paper [37], diameter distribution and aspect ratio of 70 samples of cellulose were investigated and found that the most cellulose presented diameter and aspect ratio in the range of 100–150 *μ*m and 20–25, respectively. Thus cellulose from durian peel had an aspect ratio superior to this value.

### 3.2. Tensile Strength and Impact Strength

The results of the 20 runs to determine the tensile strength and impact strength of composites using the internal mixer are tabulated in Table 3. The three factors tested in this study were cellulose loading, mixing temperature, and mixing time. The tensile strength of the composites ranged from 28.707 to 48.010 MPa and impact strength ranged from 1.922 to 2.944 kJ/m^2^. The highest tensile strength value was 48.010 MPa under test conditions of 35 wt.% cellulose loading, 165°C, and 15 min of mixing. The highest impact strength value was 2.944 kJ/m^2^ at 38.4 wt.% cellulose loading, 170°C, and 20 min of mixing; meanwhile, the lowest tensile strength value was 28.707 MPa under 25 wt.% cellulose loading, 165°C, and 15 min of mixing test conditions and the lowest impact strength value was 1.922 kJ/m^2^ under 21.6 wt.% cellulose loading, 170°C, and 20 min of mixing. When natural fibers are used as a reinforcing material in semicrystalline polymer matrices such as PLA, they can act as nucleating sites for crystal growth and commonly a transcrystalline layer grows from the crystalline cellulose surface [28, 42–44], which influences the mechanical properties of the composite. The statistical comparative study on tensile strength and impact strength of these composites was examined using RSM and the effects of the independent variables to the responses are discussed in the next section.

### 3.3. Model Selection and Verification of Tensile Strength and Impact Strength

The collected data was analyzed using software Design Expert 9. All the responses were analyzed using analysis of variance (ANOVA) and regression analysis for model fitting to evaluate the significance of the coefficient terms. The results are tabulated in Tables 4 through 6. The analysis of variance (ANOVA) for the quadratic model of tensile strength and impact strength are presented in Tables 4 and 5, respectively. The ANOVA demonstrated that the quadratic regression model of tensile strength was highly significant as the *F*-test had a very low probability value (*p* < 0.0001). This probability value means that there was only 0.01% chance that a “Model *F* value” of this magnitude could occur due to noise [45]. The model of impact strength was 0.0006 (*p* < 0.05) which also indicated that the model was significant; however, the lack of fit *F* value of 13.41 for tensile strength and 9.49 for impact strength implied that the lack of fits was also significant. The model, therefore, required further analysis. The goodness-of-fit of the models was further inspected using the *R* ^2^ value. The results showed that *R* ^2^ of the model for tensile strength and impact strength were 0.9621 and 0.9012, respectively. In addition, the adequate precision values for both tensile strength and impact strength were well above 4; therefore, all the response surface models had the satisfactory values. From the above analysis, it can be concluded that these models are suitable for predicting the mechanical properties-tensile strength and impact strength of cellulose from durian rind-reinforced PLA composites within the limits of the experiment.

The *p* values for each response are summarized in Table 6. It was found that the terms in the model had a significant effect on the responses. For the tensile strength of the composites, the mixing temperature and mixing time interaction (*X*_2_ *X*_3_) was not significant, while the other model terms were concluded to be significant. For impact strength of the composites, the cellulose loading (*X*_1_), mixing temperature (*X*_2_), mixing time (*X*_3_), two-level interaction of cellulose loading (*X*_1_^2^), mixing temperature (*X*_2_^2^), mixing time (*X*_3_^2^), and interaction of cellulose loading and mixing temperature (*X*_1_ *X*_2_) were all significant (*p* < 0.05). The other model terms were determined to not be significant. A higher value of regression coefficients can be directly translated to a greater effect of the independent variables on the responses [46]. Cellulose loading showed the highest regression coefficient value for both tensile strength and impact strength; therefore, it can be said that cellulose loading, compared to the other variables, had the greatest effect on tensile strength and impact strength. Moreover, the positive coefficients for the independent variables indicated a favorable effect on the mechanical properties [47]. The negative coefficients among the three independent variables indicated a partitioning favorable effect on the mechanical properties. Table 6 shows that only the main effect of cellulose loading represented favorable results on tensile strength and impact strength. The results demonstrated that the composites were best fit by the quadratic regression model for tensile strength and impact strength. The estimated models built for the tensile strength and impact strength methods are represented by (2) and (3) in terms of the coded values. It should be noted, however, that the following equations are only valid within the range of tested conditions: 15 wt.% < cellulose loading < 35 wt.%, 165°C < mixing temperature < 175°C, and 15 min < mixing time < 25 min.

For tensile strength, the model equation is as follows:

(2) Y=30.45−3.74X1−2.93X2−2.10X3+3.79X12+2.55X22+1.68X32−1.63X1X2+1.83X1X3−0.82X2X3.

For impact strength, the model equation is as follows:

(3) Y=2.01+0.22X1−0.14X2−0.11X3+0.15X12+0.12X22+0.12X32−0.12X1X2+0.029X1X3−0.031X2X3,

where *Y* is the predicted response; *X*_1_ is cellulose loading; *X*_2_ is mixing temperature; and *X*_3_ is mixing time.

**Figure 3:**
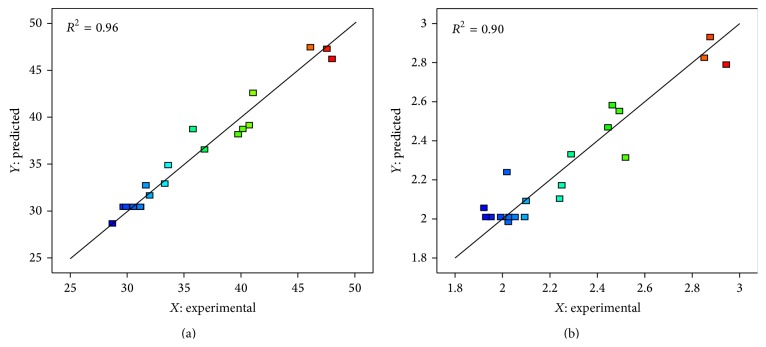
Correlation of predicted response versus experimental response: (a) tensile strength and (b) impact strength.

A graphical representation of the models' quality is shown in Figure 3. The predicted (*Y*) versus experimental (*X*) values for tensile strength (Figure 3(a)) and impact strength (Figure 3(b)) show that the quadratic model fits are suitable, with *R* ^2^ values for tensile strength and impact strength of 0.9612 and 0.9012, respectively. These *R* ^2^ values indicate that only 3.79% of the tensile strength variation and 9.88% of the impact strength variation were not explained by the models.

### 3.4. Analysis of Response Surfaces

**Figure 4:**
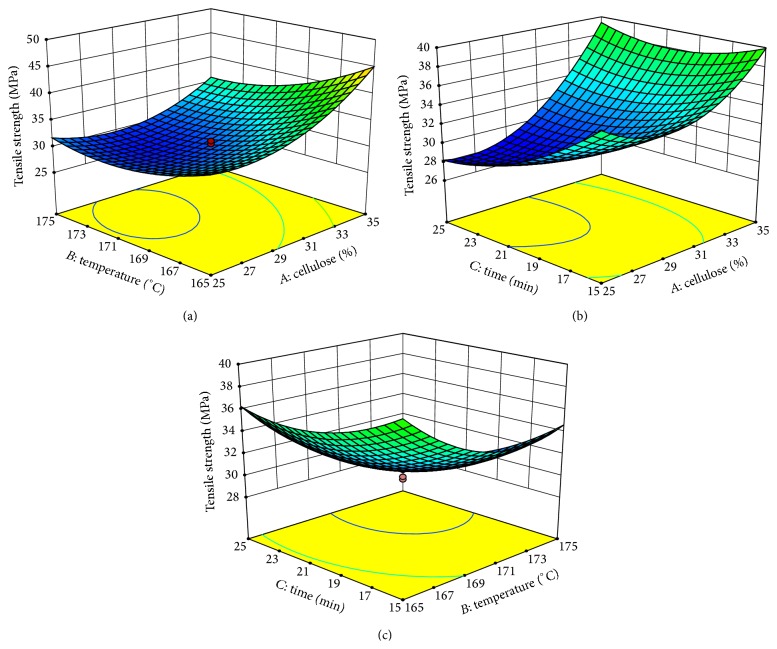
Response surfaces plots of the combined effects of the independent variables on tensile strength of biocomposites.

**Figure 5:**
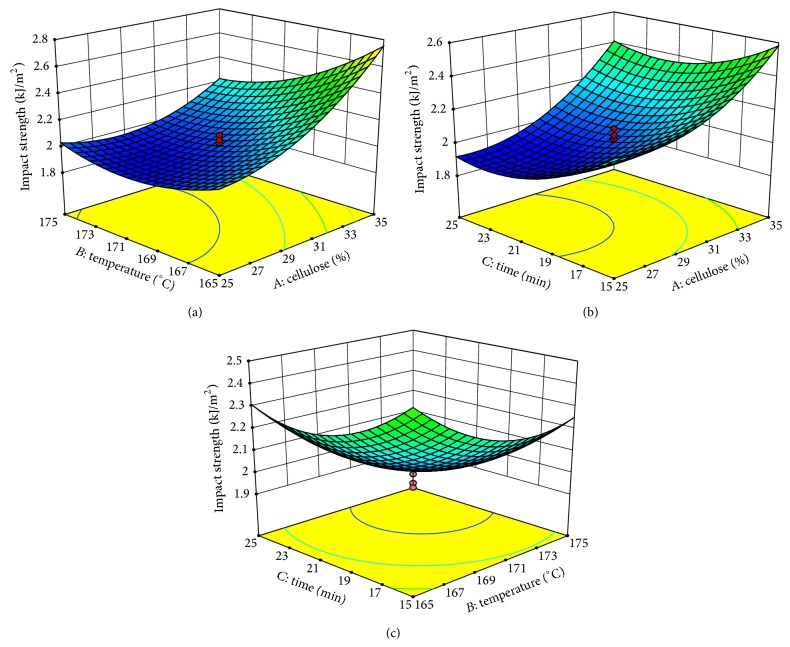
Response surface plots of the combined effects of the independent variables on impact strength of biocomposites.

The 3D response surface plots and contour plots of the combined effects of the independent variables of cellulose loading, mixing temperature, and mixing time on tensile strength and impact strength are shown in Figures 4 and 5, according to (2) and (3), respectively. In this study, 3D response surfaces were obtained by keeping one of the variables constant at a zero level while varying the other two variables. It is observed from Figure 4 that there was a quadratic effect of cellulose loading, mixing temperature, and mixing time on tensile strength.

Figures 4(a) and 4(b) demonstrate that the cellulose loading had the most significant effect on the tensile strength, followed by mixing temperature and mixing time. The tensile strength increased with increase in cellulose loading. This result was similar to the findings of Tawakkal et al. [27]. They studied the effect of kenaf derived cellulose (KDC) loading on tensile properties and reported that both the tensile strength and tensile modulus were improved with increasing KDC; the addition of KDC loading from 30 to 60 wt.% enhanced the strength of composites. In contrast, Sawpan et al. [29] investigated the mechanical properties of hemp fiber-reinforced (range 0 to 40 wt.%) PLA biocomposites and found that the relationship between tensile strength and fiber content was not linear. This indicated that at a higher fiber content, the addition somewhat decreased the strength of the composite.

Figure 4(c) illustrates that tensile strength slightly decreased with an increase in mixing temperature at a constant cellulose loading of 30 wt.%. As shown in Table 2, the highest tensile strength was obtained when the cellulose loading was at its highest level, lowest mixing temperature, and lowest mixing time within the ranges tested. The decrease in tensile strength at the higher mixing temperature and longer mixing time can be due to the thermal degradation of cellulose [48]. It is known that fiber shortening inevitably occurs during mixing of composites with both natural fiber [49, 50] and synthetic fiber [51], related to the strong shear stress endured by the viscous molten polymer [29]. As the fiber content increased, the possibility of this phenomena occurring during mixing with interaction between the fiber and equipment wall increased, resulting in fibers shorter than the critical length and reduced aspect ratio, which directly affected the performance of the composites [48] and decreased the tensile strength. Generally, the aspect ratio has to be superior to 10, which is considered to be the minimum aspect ratio for good strength transmission for any reinforcement [41]. In addition, Kannappan and Dhurai [5] reported that time did not make a significant difference in tensile strength; hence, an increase in temperature increased the tensile strength. Melting occurs when the polymer chains fall out of their crystal structures and become a disorder liquid to cause good binding results between the reinforcement fibers, which in turn increase the tensile strength.

Figures 5(a) and 5(b) present that cellulose loading had the most significant effect on the impact strength. It is clear that when cellulose loading increased, impact strength also increased rapidly at a minimum temperature as shown in Figure 5(a). According to Bledzki and Jaszkiewicz [25], the massive increase in impact performance for all man-made cellulose composites is due to smaller diameter and smoother surface, which affects the fiber/matrix interaction and, therefore, allowing for pullout to occur. The cellulose used in this study was extracted using a chlorination method to remove lignin followed by mercerization to convert holocellulose to alpha cellulose. After these two steps the noncellulosic components scattered over the surface were removed and resulted in a decreased diameter and smoother surface. Supposedly, for this reason, the path length of the propagated crack is enlarged, increasing the amount of energy needed to break the sample [52].  Figure 5(c) indicates that at a minimum mixing temperature, impact strength increased with increased mixing time, whereas at a high temperature, the longer time caused a decrease in impact strength. Tawakkal et al. [30] reported that adding kenaf derived cellulose (KDC) to the PLA matrix did not significantly contribute to the total impact strength of the composites; however, it was found that a 30 wt.% KDC/PLA composite achieved the highest impact strength, with a slightly reduced strength with the addition of 40 to 60 wt.% KDC. The decrease in impact strength indicates the lesser capacity to absorb energy under impact, due to higher crack propagation and crack initiation [53].

### 3.5. Optimization of the Experiments

**Figure 6:**
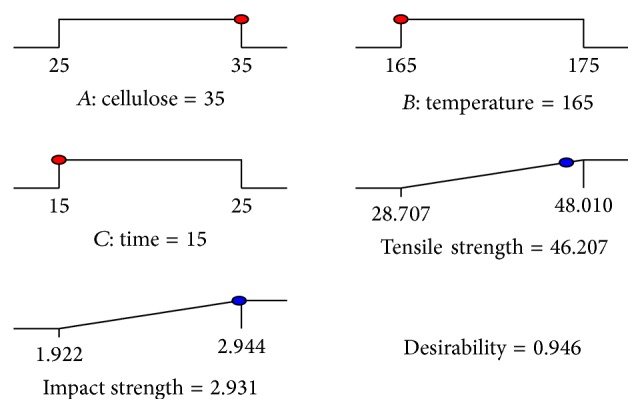
Optimum condition of the independent variables and the responses of the biocomposites.

Response surface methodology (RSM) was used to optimize the conditions for the preparation of composites. The design of experiment was carried out using Design Expert 9. In the optimization selection, there were three factors for a goal to construct desirability indices: cellulose loading, mixing temperature, and mixing time. After optimization, there were some solutions for the mechanical properties of the composites as shown in Table 6. The goal for both tensile strength and impact strength was to maximize the strengths; therefore, the target value of the responses was the highest values from the experimental results obtained. The optimization of the responses of tensile strength and impact strength is displayed in Figure 6. The acceptable values of the desirability function were the values close to one (100%). In this study, the mechanical properties of the biocomposite compromised with 35 wt.% cellulose loading, 165°C mixing temperature, and 15 min of mixing time had 94.6% desirability. These levels of the independent variables yield the highest responses of tensile strength and impact strength: 46.207 MPa and 2.931 kJ/m^2^, respectively.

This study found that the addition of cellulose, however, caused a decreasing tensile strength when compared to the neat PLA (prepared at center point with 170°C and 20 min of mixing), which produced a strength of 52.422 MPa (data not shown). Yang et al. [54] reported that an irregular shape of the reinforcement materials causes the strength of the composites to decrease due to the inability of the reinforcement to support stress transfer from the polymer matrix. A weak interfacial region will reduce the efficiency of the stress transfer from the matrix to the reinforcing component, lowering its strength [55]. The result was similar to Wang et al. [33], where they found that the strength of composites decreased with increasing amount of rice hull. A major limitation of using plant fiber and cellulose for reinforcement is that adhesion between the two materials is expected to be rather poor because of the polar nature of fiber and the nonpolar groups of thermoplastics such as poly(lactic acid) [56–58]. The quality of interfacial bonding is determined by several factors, such as the nature of the fiber and polymer components, the fiber aspect ratio, the processing procedure, and the treatment of the polymer or fiber [59–62]. To obtain materials with improved mechanical properties, the efficient dispersion of one phase into the other is required.

### 3.6. Spectroscopy Analysis

**Figure 7:**
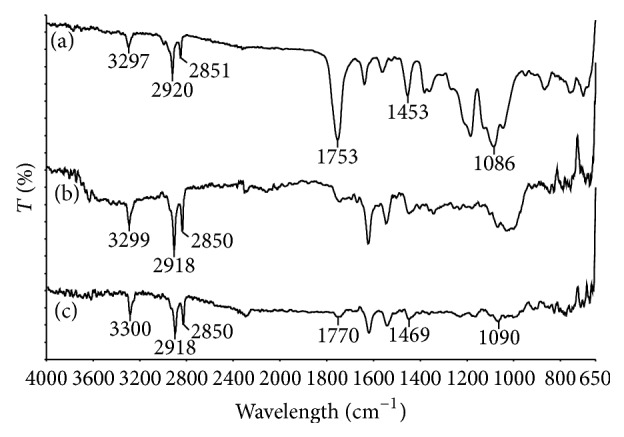
FTIR spectra for (a) poly(lactic acid), (b) cellulose, and (c) biocomposites.

The structure of polymer composites can be identified using FTIR. If two components form completely immiscible blend, then there should be no considerable changes in the IR spectra of composites compared with each component spectra [63]. However, if two polymers are compatible, a distinct chemical interaction (hydrogen-bonding or dipolar interaction) exists between the chains of polymer and those of additional component, affecting the IR spectra of composites to change (e.g., band shifting and broadening) [64].  Figure 7 shows the spectra of PLA, cellulose and biocomposites (PLA/cellulose blends). Peaks near 3400–3200 cm^−1^ and 3000–2850 cm^−1^ were observed in all spectra which are attributed to O-H stretching group [37] and C-H stretching [65], respectively. For neat PLA, the strong absorption peak at 1753 cm^−1^ is assigned to stretching of C=O group [27, 63, 66] and peak at 1080 and 1453 cm^−1^ represents C-O group of ester bonds and asymmetrical stretching of -CH_3_ group [66]. It was seen that there were shifts of C=O peak at 1753 cm^−1^ and C-O peak at 1086 cm^−1^ (neat PLA) to peak at 1770 cm^−1^ and 1090 cm^−1^ in biocomposites. These shifts might be because of the formation of hydrogen bonding between -OH in cellulose and C=O and C-O in PLA [27, 30, 67]. The peak at 1453 cm^−1^ (neat PLA) moved toward higher wavenumber of 1469 cm^−1^ in biocomposites. The observations further indicated the presence of interactions between the PLA matrix and cellulose surfaces.

## 4. Conclusion

The biocomposites showed that tensile strength ranged from 28.707 to 48.010 MPa and impact strength ranged from 1.922 to 2.944 kJ/m^2^. The highest tensile strength value was 48.010 MPa with conditions of 35 wt.% cellulose loading, 165°C, and 15 min of mixing, while the highest impact strength value was 2.944 kJ/m^2^ under 38.4 wt.% cellulose, 170°C, and 20 min of mixing. The quadratic regression model was selected for modeling the tensile strength and impact strength due to its high significance level. The *R* ^2^ values of the model for tensile strength and impact strength were 0.9621 and 0.9012, respectively. Both tensile strength and impact strength had adequate precision values above 4; thus, these models can be used to predict the mechanical properties of biocomposites composed of poly(lactic acid) and cellulose from durian rind. Cellulose loading, mixing temperature, and mixing time were significant variables in affecting tensile strength and impact strength. The order of independent variables according to the significance was cellulose loading followed by mixing temperature and mixing time. The optimal conditions considering the mechanical properties was found to be at cellulose loading, mixing temperature, and mixing time of 35 wt.%, 165°C, and 15 min with a desirability of 94.16%. At this optimal condition, the tensile strength and impact strength were found to be 46.207 MPa and 2.931 kJ/m^2^, respectively.

## Figures and Tables

**Table 1:** Coded levels of variables.

Variables	Coded levels				
	−*α*	−1	0^a^	+1	+*α*
*X*_1_: cellulose loading (wt. %)	21.6	25	30	35	38.4
*X*_2_: temperature (°C)	161.6	165	170	175	178.4
*X*_3_: time (min)	11.6	15	20	25	28.4

^a^Center point; *k* = 3 (three independent variables).

*α* = 1.6818.

**Table 2:** The arrangement of the central composite design.

Trial	Coded variables			Actual variables		
	*X*_1_	*X*_2_	*X*_3_	*X*_1_ (%)	*X*_2_ (°C)	*X*_3_ (min)
1	−1	−1	−1	25	165	15
2	−1	−1	+1	25	165	25
3	−1	+1	−1	25	175	15
4	−1	+1	+1	25	175	25
5	+1	−1	−1	35	165	15
6	+1	−1	+1	35	165	25
7	+1	+1	−1	35	175	15
8	+1	+1	+1	35	175	25
9	−*α*	0	0	21.6	170	20
10	+*α*	0	0	38.4	170	20
11	0	−*α*	0	30	161.6	20
12	0	+*α*	0	30	178.4	20
13	0	0	−*α*	30	170	11.6
14	0	0	+*α*	30	170	28.4
15	0	0	0	30	170	20
16	0	0	0	30	170	20
17	0	0	0	30	170	20
18	0	0	0	30	170	20
19	0	0	0	30	170	20
20	0	0	0	30	170	20

**Table 3:** The responses of the parameters used in central composite design.

Trial	Independent variables			Responses	
	Cellulose loading (%)	Temperature (°C)	Time (min)	Tensile strength (MPa)	Impact strength (kJ/m^2^)
1	25	165	15	40.735 ± 1.184	2.519 ± 0.510
2	25	165	25	33.318 ± 0.817	2.010 ± 0.122
3	25	175	15	39.762 ± 1.092	2.290 ± 0.310
4	25	175	25	28.707 ± 1.231	2.025 ± 0.248
5	35	165	15	48.010 ± 1.208	2.876 ± 0.356
6	35	165	25	47.556 ± 1.669	2.851 ± 0.227
7	35	175	15	40.174 ± 0.663	2.445 ± 0.131
8	35	175	25	36.800 ± 1.380	2.019 ± 0.538
9	21.6	170	20	33.615 ± 1.461	1.922 ± 0.171
10	38.4	170	20	46.114 ± 0.956	2.944 ± 0.341
11	30	161.6	20	41.067 ± 1.901	2.464 ± 0.297
12	30	178.4	20	31.662 ± 1.146	2.241 ± 0.540
13	30	170	11.6	35.793 ± 1.076	2.493 ± 0.196
14	30	170	28.4	31.984 ± 1.048	2.250 ± 0.289
15	30	170	20	29.920 ± 2.051	2.053 ± 0.210
16	30	170	20	30.658 ± 1.483	1.952 ± 0.252
17	30	170	20	31.150 ± 1.286	1.930 ± 0.250
18	30	170	20	30.549 ± 0.845	1.993 ± 0.165
19	30	170	20	29.666 ± 1.615	2.028 ± 0.153
20	30	170	20	31.192 ± 1.501	2.094 ± 0.203

**Table 4:** Analysis of variance for the quadratic model of tensile strength.

Source	Sum of squares	DF	Mean squares	*F* value	Prob >*F*
Model	715.11	9	79.46	28.18	<0.0001 significant
Residual	28.20	10	2.82		
Lack-of-fit	26.24	5	5.25	13.41	0.0064 significant
Pure error	1.96	5	0.39		
Total	743.31	19			

Std. dev. = 1.68, mean = 35.92, *R*-square = 0.9621, and adeq. precision = 15.814.

**Table 5:** Analysis of variance for the quadratic model of impact strength.

Source	Sum of squares	DF	Mean squares	*F* value	Prob >*F*
Model	1.84	9	0.20	10.13	0.0006 significant
Residual	0.20	10	0.020		
Lack-of-fit	0.18	5	0.037	9.49	0.0137 significant
Pure error	0.019	5	3.850*E* − 003		
Total	2.04	19			

Std. dev. = 0.14, mean = 2.27, *R*-square = 0.9012, adeq. precision = 9.415.

**Table 6:** Regression coefficients and probability values of approximate polynomials for response variables in experimental design.

Term	Tensile strength		Impact strength	
	Coefficient	Probability	Coefficient	Probability
Constant	30.45	—	2.01	—
*X*_1_: cellulose	3.74	<0.0001	0.22	0.0002
*X*_2_: temperature	−2.93	<0.0001	−0.14	0.0041
*X*_3_: time	−2.10	0.0009	−0.11	0.0148
*X*_1_^2^	3.79	<0.0001	0.15	0.0029
*X*_2_^2^	2.55	0.0002	0.12	0.0103
*X*_3_^2^	1.68	0.0036	0.12	0.0076
*X*_1_ *X*_2_	−1.63	0.0209	−0.12	0.0382
*X*_1_ *X*_3_	1.83	0.0116	0.029	0.5752
*X*_2_ *X*_3_	−0.82	0.1975	−0.031	0.5538
